# Quantitative stability of CT numbers in novel cone‐beam CT: An intra‐institution cross‐scanner and longitudinal analysis

**DOI:** 10.1002/acm2.70752

**Published:** 2026-08-24

**Authors:** Vicki Trier Taasti, Mai‐Britt Linaa, Camilla Skinnerup Byskov, Morten Bjørn Jensen, Ditte Sloth Møller, Lone Hoffmann

**Affiliations:** ^1^ Danish Centre for Particle Therapy Aarhus University Hospital Aarhus Denmark; ^2^ Department of Clinical Medicine Aarhus University Aarhus Denmark; ^3^ Department of Oncology Aarhus University Hospital Aarhus Denmark

**Keywords:** CBCT dose calculation, CT number stability, Novel CBCT imaging

## Abstract

**Background:**

Novel cone‐beam computed tomography (CBCT) has become available on standard C‐arm linacs, making it widely applicable in photon‐based radiotherapy.

**Purpose:**

To evaluate CT number stability of novel CBCT systems installed on seven C‐arm linacs within a single institution, across CBCT systems and over time, and to assess the CBCT‐based dose calculation accuracy in head‐and‐neck, lung, and pelvic cancer patients.

**Methods:**

A Gammex Advanced Electron Density phantom (Sun Nuclear) was scanned at installation of eleven CBCT imaging panels at seven TrueBeam C‐arm linacs (Varian Medical Systems, A Siemens Healthineers Company), and after three and six months. Three CBCT protocols were evaluated, a head protocol (tube voltage of 100 kVp, iterative reconstruction, denoted iCBCT), a thorax protocol (tube voltage of 125 kVp; filtered back projection with Feldkamp‐Davis‐Kress (FDK) algorithm), and a pelvis protocol (tube voltage of 125 kVp; iCBCT).

Fifteen tissue‐equivalent phantom inserts were scanned individually, placed centrally in the phantom. The mean CT number was extracted, and CT number stability was assessed across imaging panels and timepoints. CT number stability was compared to a previous CBCT detector model. Moreover, routine measurements using four inserts were performed until 18 months after installation. Conversion curves for mass density estimation were generated, following a consensus guide. The spread of the curves due to CT number variation across imaging panels was assessed.

Treatment plans created on planning CT (pCT) scans of 10 head‐and‐neck, 22 lung, and 15 pelvic cancer patients were recalculated on CBCT scans, selected to match the anatomy seen on the pCT. Dose‐volume parameters for targets and organs‐at‐risk were compared between pCT and CBCT.

**Results:**

The CT numbers were consistent across the imaging panels and over time for iCBCT reconstruction, but to a lesser degree for FDK reconstruction. The CT number variation increased with the density of the phantom inserts. Still, for the high‐density bone insert the range/interquartile range of the CT numbers across imaging panels were 51/29 HU (head protocol), 140/41 HU (thorax protocol), and 27/16 HU (pelvis protocol). For the longitudinal measurements, the median differences were 4 HU, ‐9 HU, and ‐8 HU after three months and ‐8 HU, ‐32 HU, and ‐26 HU after six months, for the head, thorax, and pelvis protocol, respectively.

For the routine measurements, no clear time dependence was seen for the CT number differences. It was found that a single conversion curve per CBCT protocol could be used for all imaging panels. For iCBCT, median dose differences between pCT and CBCT were within 0.5% for head‐and‐neck and pelvis and 1.5% for lung, while FDK lung was within 2.5%. The largest deviations with iCBCT were ‐1.3% for head‐and‐neck, ‐1.1% for pelvis, and ‐3.8% for thorax, but ‐18.0% for thorax FDK.

**Conclusions:**

The CT number stability was sufficient to allow for a single conversion curve per CBCT protocol to be applied across all CBCT imaging panels. A high dose calculation accuracy was found for 36 patients with iCBCT scans, while larger deviations were seen for eleven thorax FDK scans.

## 1 INTRODUCTION

In many patient populations, adaptive radiotherapy is important to ensure consistent target coverage over the full course of treatment.^1^, ^2^, ^3^ The prerequisite for adaptive radiotherapy is to have a way of triggering when a plan adaptation is needed.^4^ A good trigger is dose recalculation, as this allows for evaluating the dose distribution on the daily anatomy in the same way as the original plan was judged, namely based on dose‐volume histogram (DVH) parameters and/or visual inspection of the dose distribution.^5^

However, dose recalculation based on the daily anatomy requires repeated imaging, either using computed tomography (CT) or cone‐beam CT (CBCT).^6^ As CBCT is often used for patient alignment, it would not impose additional dose to the patient, if these CBCT scans could also be used for dose re‐evaluation.^4^, ^7^, ^8^ However, conventional CBCT images are affected by increased scatter, image artifacts, and inaccurate CT numbers which might drift over time, and this can compromise the accuracy and robustness of dose calculations used in adaptive radiotherapy.^9^ Several methods have been established to account for this and enable accurate CBCT‐based dose calculation.^10^, ^11^ New approaches involving deep learning have also been developed to improve the image quality.^12^, ^13^

Fortunately, newer CBCT scanners, like the HyperSight CBCT system, include better detectors, ensuring higher stability as well as advanced reconstruction algorithms, reducing the image noise and image artifacts.^14^ This ensures higher CT number accuracy, and it has been shown that these CBCT images can be used directly for photon dose recalculation with high accuracy without applying any further image quality improvement algorithms.^15^, ^16^, ^17^, ^18^ Recently, the CT number stability was evaluated across HyperSight CBCT systems in a multi‐center study including six institutions, showing low CT number variability across scanners for relative electron densities below 1, whereas a larger spread was seen for dense materials.^19^ Additionally, a recent longitudinal assessment of the CT number stability based on CBCT scans of a prostate cancer cohort acquired over four months showed high temporal stability.^20^ These improved CBCT reconstruction algorithms have even passed most of the criteria set out for diagnostic CT imaging.^21^ However, large‐scale stability data are currently unavailable, which limits the ability to ensure accurate and reliable dose calculations over the multi‐year period during which a CBCT scanner is in clinical operation. All these studies were conducted on O‐ring linear accelerators (linacs), whereas the new CBCT technology is now also available for C‐arm linacs.^22^, ^23^

The aim of this study was to assess the dose calculation accuracy directly based on HyperSight CBCT images without correction methods. The CT number stability across imaging panels and over time was thus evaluated for CBCT systems installed on seven C‐arm linacs at a single institution, to assess whether universal conversion curves for mass density estimation could be derived. Applying the conversion curves, we investigated the accuracy of dose calculations on CBCT images for 47 head‐and‐neck, lung, and pelvic cancer patients, encompassing a variety of anatomical sites.

## 2 METHODS

### 2.1 CBCT scanning and reconstruction settings

A Gammex Advanced Electron Density phantom (Sun Nuclear—A Mirion Medical Company, Middleton, WI, USA) was scanned with HyperSight CBCT systems (Varian Medical Systems—A Siemens Healthineers Company, Palo Alto, CA, USA) installed on seven TrueBeam linacs (Varian) in one institution. CBCT scans were acquired at the time of installation, as well as after 3 and 6 months. For some of the CBCT systems, the imaging panel was exchanged due to breakdown (at two linacs, the imaging panel was replaced once, and at one linac, it was replaced twice). This resulted in a total of eleven scanning sessions at installation, eight scanning sessions at 3 months, and seven (one at each linac) at 6 months. For each scanning session, the phantom was aligned with the in‐room lasers, to ensure consistent alignment.

At each timepoint, the phantom scans were performed with three CBCT scan protocols to cover the range of scan protocols used for patient scanning, namely a head protocol, thorax protocol, and pelvis protocol. For all three scan protocols, the default scan and reconstruction settings were used. These included a tube voltage of 100 kVp for the head protocol, and 125 kVp for the thorax and pelvis protocols. A tube current of approximately 150, 270, and 1070 mAs and a CBCT scan time of 23 s, 40 s, and 40 s were applied for the head, thorax, and pelvis protocols, respectively. For the head and pelvis protocols, iterative reconstruction (denoted iCBCT) was used, while standard filtered back‐projection reconstruction applying the Feldkamp‐Davis‐Kress (FDK) algorithm^24^ was used in the thorax protocol. The slice thickness for all reconstructed CBCT images was 2.5 mm.

For the head protocol, the small inner part of the phantom (round, 20 cm diameter) was scanned, while for the thorax and pelvis protocols, the full phantom (elliptical, 30 × 40 cm) was scanned. During CBCT scanning, the phantom was placed directly on the treatment table and not in the phantom holder, to be able to place three solid water blocks (Gammex, Middleton, WI, USA) at each end of the phantom to provide sufficient scatter material. Each solid water block had a thickness of 5 cm, and the phantom itself was 16.5 cm long. The feasibility of CBCT scanning without the solid water blocks was initially evaluated; however, superior CT number stability was achieved when the blocks were added. The full CBCT scan length was 25.4 cm, but it was collimated to 20 cm.

For each of the three CBCT protocol, nineteen CBCT acquisitions were performed for different phantom insert configurations (see Table 1). Each insert configuration contained one tissue‐equivalent insert in the middle of the phantom, while the remaining fifteen insert holes were filled with HE Solid Water inserts. Only the CT number of the insert placed in the middle of the phantom was used for evaluation. A total of 15 different inserts (8 soft tissue, and 7 bone inserts) were included, with mass densities ranging from 0.289 to 1.775 g/cm^3^.

**TABLE 1:** Scan order of the phantom inserts (each phantom insert placed centrally in the phantom and was scanned separately) and the intent of each scan. A forced dark field measurement was performed between scan 1 and 2. The listed mass density is the batch specific mass density specified on the phantom certificate. Note, two individual Lung LN450 inserts were included, they had the same elemental composition but different mass densities. Also note that the three bone inserts, Inner Bone, B‐200, and Cortical Bone, have a chemical composition corresponding to the inserts of the predecessor Gammex 467 phantom, but they have the same 16.5 cm length as the other inserts.

Scan number	Scan intent	Insert name	Mass density (g/cm^3^)
1	Dark field	HE Cortical bone	1.775
2	Dark field / After‐glow	HE Cortical bone	1.775
3	After‐glow	HE Solid Water	0.997
4	CT number stability	Lung LN300	0.289
5	CT number stability	Lung LN450 A	0.454
6	CT number stability	Lung LN450 B	0.483
7	CT number stability	HE General Adipose	0.950
8	CT number stability	HE Breast 50:50	0.971
9	CT number stability / After‐glow	HE Solid Water	0.997
10	CT number stability	HE Brain	1.050
11	CT number stability	HE Liver	1.053
12	CT number stability	Inner Bone	1.100
13	CT number stability	B‐200	1.109
14	CT number stability	HE Inner Bone	1.167
15	CT number stability	CaCO_3_ 30%	1.266
16	CT number stability	CaCO_3_ 50%	1.460
17	CT number stability	Cortical Bone	1.687
18	CT number stability	HE Cortical Bone	1.775
19	After‐glow	HE Solid Water	0.997

### 2.2 Evaluation of potential CBCT system artefacts

The effect of acquiring a forced dark field measurement (acquisition without turning the X‐ray source on, to measure the intrinsic signal of the detector elements^25^) prior to CBCT scanning was examined. Two CBCT scans of the phantom, each with the HE Cortical Bone insert positioned in the center, were acquired, one before and one after acquiring a forced dark field. The resulting CT number difference between the two CBCT scans was quantified.

To investigate whether after‐glow^26^ (signal from previous CBCT scans) influenced the CBCT scans, the HE Cortical Bone and the HE Solid Water inserts were scanned repeatedly throughout the measurement series (see Table 1), to assess whether a CT number drift occurred over the measurement series. The HE Cortical Bone insert was scanned twice after the forced dark measurement, once at the start of the measurement series and once its end, while the HE Solid Water insert was scanned three times, at the start, the middle, and at the end of the measurement series. Each measurement series comprised 19 CBCT scans and lasted, on average, 41 min (range: 25–97 min).

### 2.3 CT number stability

To assess if accurate and consistent dose calculation could be performed based on the CBCT images, we evaluated the CT number stability, since if a CT number drift would be seen this would affect the dose calculation accuracy or require repeated re‐calibration of the conversion curves.

The mean CT number was extracted for the insert placed in the middle of the phantom, applying a region‐of‐interest with a radius of 9 mm in‐plane and 2 cm in the z‐direction with the region‐of‐interest centered in the middle of the insert. The CT number variation across CBCT imaging panels was evaluated for each insert for the measurement series acquired at installation of the imaging panel. The CT number stability over time was assessed as the CT number difference between the CBCT measurement at installation and the CBCT measurement after three or six months for each imaging panel. In total, 78 measurement series were performed, each containing 15 CT number measurements. Thereafter, CT number stability checks were performed every third month based on a limited set of phantom inserts (Lung LN300, HE Solid Water, CaCO_3_‐50%, and HE Cortical Bone), but still with each insert scanned separately, placed in the center of the phantom. This was performed as part of the routine quality assurance (QA) checks.

The CT number stability for the HyperSight CBCT system was compared to the CT number stability measured for the previous non‐HyperSight CBCT system installed at the same linacs (Varian TrueBeam v. 2.7, Varian Medical Systems). The non‐HyperSight CBCT system consisted of a different detector material^9^, ^22^ and only offered FDK reconstruction. For these former CBCT systems, CBCT data acquired in the same way as in this study only existed for a single time point (after several years of use). These data were compared to the HyperSight data acquired at installation.

### 2.4 Mass density conversion curves

In this study, we applied an updated version of a guide on generation of CT conversion curves^27^ for converting CT numbers to mass density. For each CBCT protocol (head, thorax, and pelvis), one conversion curve was created based on the CT numbers measured at the installation, with the CT number for each phantom insert averaged over all imaging panels. Additionally, a conversion curve was generated using the CT numbers for each phantom insert averaged over all CBCT acquisitions (at installation, after 3 and 6 months, and all CBCT imaging panels), as well as conversion curves for the average ± 1 or 2 standard deviations (SD) of the CT numbers. These were used to evaluate whether a single conversion curve for each CBCT protocol was acceptable, rather than requiring individual calibration for each CBCT system.

### 2.5 Dose calculation in patients

To evaluate the dose calculation accuracy based on the CBCT images, clinical treatment plans for ten head‐and‐neck (HN) cancer patients, twenty‐two lung cancer patients, and fifteen pelvic cancer patients were recalculated on a CBCT acquired before treatment delivery. For the HN and pelvic cancer patients, treatment plans used the volumetric modulated arc therapy (VMAT) delivery technique, while the lung cancer patients were planned with intensity‐modulated radiation therapy (IMRT). All patients were treated and scanned in free breathing. For all patients, the treatment plan was created on a fan‐beam CT scan, acquired at either a Philips GEMINI TF Big Bore PET/CT scanner or a Siemens SOMATOM go.Open Pro CT scanner. All fan‐beam CT scans were acquired at 120 kVp. The conversion curves for the fan‐beam CT scans were created in the same way as for the CBCT described above (calibrated individually for each CT scanner). For the lung cancer patients, a 4DCT was acquired, and the mid‐ventilation phase (chosen visually based on the breathing trace, using amplitude‐based phase binning) was used for treatment planning.

For each patient, a single CBCT image was chosen for this evaluation, selected to match the anatomy seen on the planning fan‐beam CT as closely as possible. Since the planning CT image for the lung cancer patients was a single 4DCT phase, whereas the CBCT image represents a breathing‐averaged image due to the scan time of 40 s, only lung cancer patients with a relatively small tumor motion amplitude were included, to ensure a consistent tumor position between the CT and CBCT images and avoid tumor blurring on the CBCT image.

For the HN and pelvic cancer patients, the CBCT images were reconstructed with the iterative iCBCT algorithm. For the first eleven lung cancer patients, the CBCT images were reconstructed with the FDK algorithm according to the vendor's recommendation. However, the calculated dose distributions for these lung cancer patients were found to differ systematically from the dose distributions on the planning CT. Therefore, eleven new lung cancer patients were included. For these patients, the pelvis mode was applied (with a reduced mAs similar to the thorax mode), whereby the CBCT images were reconstructed with the iterative iCBCT algorithm.

The target and organ‐at‐risk (OAR) structures were copied from the planning CT to the CBCT image in MIM (MIM Software, Cleveland, OH) using deformable image registration for the HN and lung cancer patients and rigid registration for the pelvic cancer patients. The deformed structures were checked as part of the patient selection, and the final set of 47 patients included in the evaluation therefore had proper deformed structures. In patients where only a single structure deviated due to anatomical changes in that region while the rest of the anatomy looked correct, this structure was excluded from the evaluation.

For the dose recalculation on the CBCT images, the conversion curve generated based on the CT numbers averaged over all imaging panels at installation was applied. The clinical treatment plan was Monte Carlo recalculated using SciMoCa® (Scientific RT, Munich, Germany)^28^, ^29^ on both the planning CT and the CBCT, to have consistent dose calculation. Selected DVH parameters were extracted from the planning CT and the CBCT scans to compare the doses with respect to target coverage (D98%), target mean doses, and near maximum doses (D1cc) to relevant OARs. The DVH differences were calculated relative to the prescription dose for the individual patient, as the prescription dose varied among the patients. The patient description and the prescription doses can be found in Tables S1–S3 in the supplementary material.

## 3 RESULTS

### 3.1 Effect of dark field measurement and detector after‐glow

The effect of performing a forced dark field acquisition was minimal for the head and pelvis CBCT protocols applying the iCBCT reconstruction, with median CT number differences between the CBCT scans acquired before and after the dark field acquisition of 2.8 HU and 1.0 HU, respectively (Figure S1 in the supplementary material). The CT number differences were all within ± 35 HU and ± 29 HU, with inter‐quartile ranges (IQRs) of 7.3 HU and 9.4 HU, for the head and pelvis protocols, respectively. Larger CT number differences were seen for the thorax protocol with the FDK reconstruction, with a median CT number difference of ‐6.2 HU and IQR of 52.5 HU, with CT number difference up to 80 HU (excluding one outlier value of 232 HU).

The CT numbers for HE Cortical Bone and HE Solid water remained consistent over the measurement series, showing minimal after‐glow (Figure S2 in the supplementary material). The smallest CT number differences were seen for the HN scan protocol, while the largest differences were seen for the thorax protocol, but in all cases, the median CT number difference was close to zero. The median CT number differences and IQRs were within 12.6 HU and 23.5 HU for the HE Cortical Bone insert. For the HE Solid water insert, they were within 2.5 HU and 12.1 HU. This indicated that it was acceptable to perform the full measurement series with 19 scans without breaks. This ensured the feasibility of the conversion curve calibration procedure with individual CBCT measurements for each insert.

### 3.2 CT number variations

**FIGURE 1:**
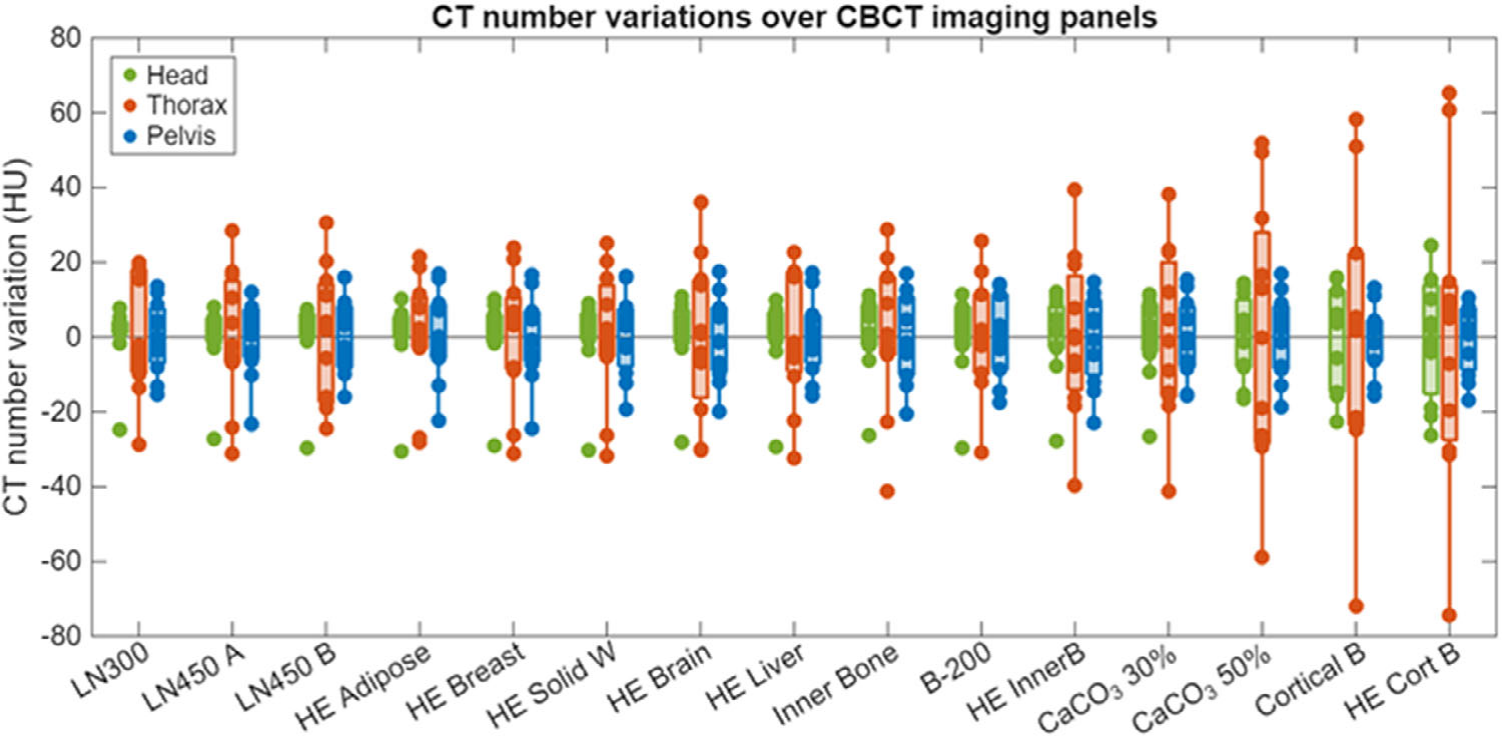
CT number variation across the CBCT imaging panels measured at the installation. The data is shown as the mean over all imaging panels minus the actual CT number for the specific imaging panel for each of the 15 inserts. For the boxplots, the boxes cover the interquartile range (IQR; 25^th^ to 75^th^ percentile), and the whiskers extend to 1.5 times the IQR. The data is plotted for each of the three CBCT protocols (Head, green; Thorax, red; and Pelvis, blue) and each insert. The x‐axis labels refer to the (abbreviated) name of the insert. ‘LN450 A/B’ are two different inserts with different mass density; ‘HE Solid W’ is ‘HE Solid Water’; ‘HE InnerB’ is ‘HE Inner Bone’; ‘Cortical B’ is ‘Cortical Bone’; ‘HE Cort B’ is ‘HE Cortical Bone’. Note: Insert names with and without ‘HE’ refers to different inserts with different chemical composition and density.

The largest CT number variation across the CBCT imaging panels measured at installation was found for the thorax protocol, which included FDK reconstruction (Figure 1). The CT number variation increased with the density of the phantom inserts, especially for the thorax protocol. For the HE Cortical Bone insert, which had the highest mass density, the IQR of the CT number variation across the imaging panels was 29 HU for the head protocol, 41 HU for the thorax protocol, and 16 HU for the pelvis protocol. The results for the CT number variation were comparable between the HyperSight and the previous non‐HyperSight CBCT system (Figure S3 in the supplementary material).

**FIGURE 2:**
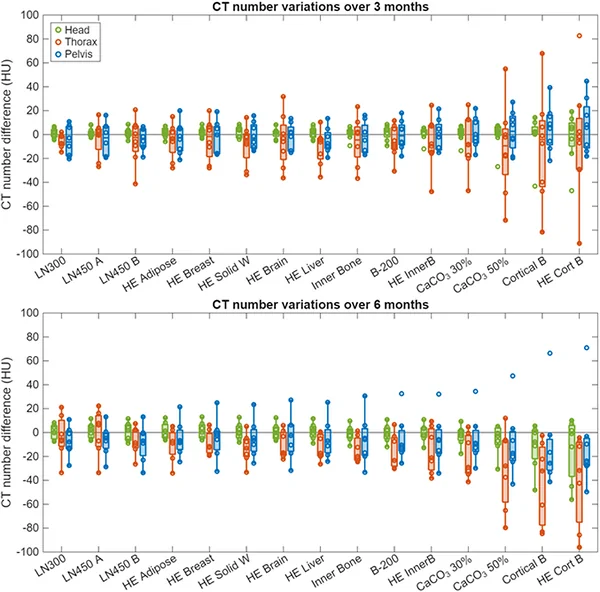
CT number difference for each specific CBCT imaging panel measured three (top) or six (bottom) months after installation. The CT number difference was defined as the measured CT number at 3 or 6 months minus the measured CT number at installation. See caption for Figure 1 for further information.

The CT number difference between the measurements at installation and after three and six months only showed small differences (Figure 2). Again, the highest differences were seen for the thorax protocol. For each insert, the largest (in either positive or negative direction) median differences were 4 HU, ‐9 HU, and ‐8 HU after 3 months and ‐8 HU, ‐32 HU, and ‐26 HU after 6 months, for the head, thorax, and pelvis protocol, respectively. This shows a general tendency towards lower CT numbers over time, but with only a small CT number drift. However, for the pelvis mode at the 6‐months assessment, there was one set of outlier values showing the opposite behavior, i.e. increasing CT numbers (positive differences). These outlier values, seen especially for the high‐density inserts, all belonged to the same imaging panel. To deal with this, this CBCT imaging panel was first recalibrated, which did not correct the behavior. Next, the CBCT detector was turned off and on again, which had a positive impact on the CT number differences. For the HE Cortical Bone insert, the CT number difference decreased from 71 HU to 39 HU (Figure S4 in the supplementary material).

For the routine QA measurements with a limited set of inserts, no clear trend was visible for the CT number differences against time (Figure S5 in the supplementary material). The median of the absolute CT number differences for the HE cortical bone insert were 12 HU, 32 HU, and 18 HU during a time period of up to 18 months after installation, for the head, thorax, and pelvis protocol, respectively.

### 3.3 Mass density conversion curves

The mass density conversion curves, calibrated based on the average across all the measurements at installation, as well as average ± SD across all measurements (installation, 3 months, and 6 months), are shown in Figure S6 in the supplementary material. For the head and pelvis protocol, a small variability was observed in the adipose region; however, in the bone region, where the largest variability would be expected due to the larger CT number variation in this region, minimal variation was seen between the conversion curves. For the head protocol, the mass density spread at a CT number of 1125 HU (medium dense bone, mass density of ∼1.6 g/cm^3^) was 0.02 between the conversion curves based on the average CT numbers minus 2SD and the average CT numbers plus 2SD. This is a variation of 1.5% relative to the mass density estimated using the conversion curve for the averaged CT numbers across all measurements. For the pelvis protocol, the deviation between these two extreme curves at a CT number of 800 HU (corresponding to a mass density of ∼1.6 g/cm^3^) was 0.03 (2.2% relative to the mass density estimated using the conversion curve for the averaged CT numbers across all measurements). Different CT numbers were used in this evaluation, because of the different tube potentials: 100 kVp for the head protocol and 125 kVp for the pelvis protocol. The small differences between the extreme curves indicated that a single conversion curve could be applied for each specific protocol, regardless of which CBCT system was being used, which was exploited in the dose recalculations for the patients.

Furthermore, for these two protocols, the conversion curve calibrated based on the average CT numbers across the different detectors measured at installation and the conversion curve calibrated based on the average CT numbers, including all time points, were nearly inseparable, showing that the small CT number drift did not impact the conversion curves.

For the thorax protocol, almost no variation was observed between the individual curves in the adipose region, but in the bone region, a deviation was clearly visible. At a CT number of 800 HU, the deviation between the two extreme curves was 0.08 (5.1%). Moreover, a small, visible separation was observed between the average curves calibrated only based on data from installation and curves calibrated based on data from all timepoints, showing the impact of the CT number drift over time.

### 3.4 Dose calculations in patients

**FIGURE 3:**
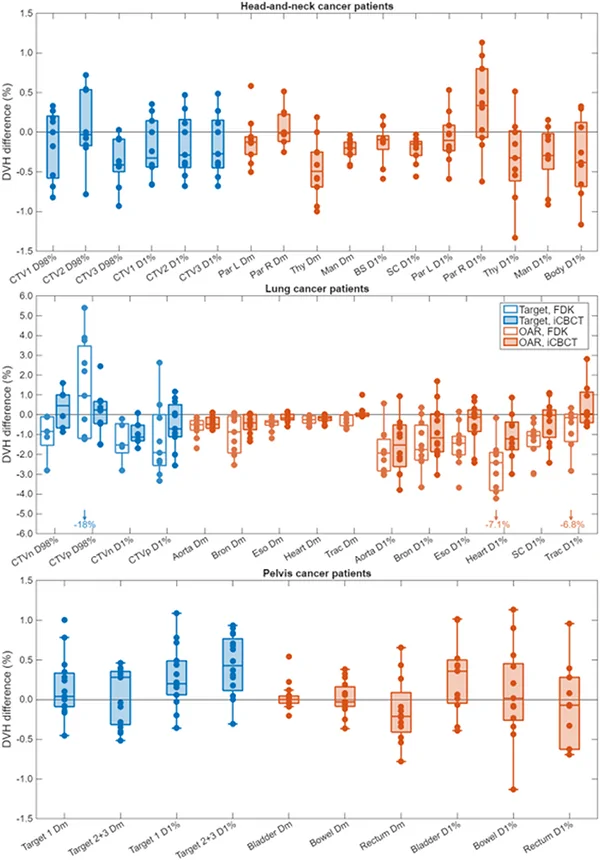
DVH differences between the treatment plan calculated on the planning CT and on the CBCT, relative to the prescription dose (ΔDVH = (DVH(CT)‐DVH(CBCT))/Dp; Dp: prescription dose). Blue boxplots for the target metrics, and orange boxplots for the OAR metrics. The filled boxplots represent CBCTs reconstructed with iCBCT, while the non‐filled boxplots for the lung cancer patients represent CBCTs reconstructed with FDK (the CBCT images reconstructed with iCBCT and FDK correspond to different patients). Note, the different y‐axis scales. Also, to better visualize the data for the lung cancer patients, three outlier values for the FDK reconstruction are not shown but indicated with arrows: for CTVp D98% at ‐18.0%, Heart D1% at ‐7.1%, and Trachea D1% at ‐6.8%. Abbreviations: Dm: mean dose, Par L/R: Parotid left/right, Thy: Thyroid, Man: Mandible, BS: Brainstem, SC: Spinal cord, Bron: Bronchi, Eso: Esophagus, Trac: Trachea.

The DVH differences between the dose on the planning CT and the anatomically matched CBCT are shown in Figure 3. For the HN and pelvic cancer patients, the median dose differences were within ± 0.5%, while most dose differences were within ± 1%, with a maximum difference of ‐1.3%. However, for the lung cancer patients with CBCT scans reconstructed with FDK, larger deviations were observed, up to ‐18.0% (outlier values not shown), and median deviations up to ± 2.4%. Due to the substantial dose deviations observed for these CBCTs, which were not observed in HN or pelvic cancer patients with CBCTs reconstructed using iCBCT, additional lung cancer patients were included for whom the pelvis protocol, and thus iCBCT reconstruction, was applied.

For these CBCTs, the median deviations were generally closer to zero compared to the CBCTs reconstructed with FDK, and for some DVH parameters, the spread across the patients was also narrower. The median deviations were within ± 1.5%, with maximum deviations of ± 3% except for one deviation of ‐3.8%. However, the dose differences for lung cancer patients, even when reconstructed with iCBCT, were still larger than for HN and pelvic cancer patients. This may be explained in part by the respiratory motion in this anatomical region.

## 4 DISCUSSION

In this study, we evaluated the dose calculation accuracy of HyperSight CBCT systems installed at seven TrueBeam linacs in a single institution, by assessing the CT number stability across CBCT imaging panels and over an extended time period of 18 months. We found that the biggest gain regarding stability from this new technology was the iterative reconstruction (iCBCT). The CT number variance across the CBCT imaging panels was lowest for the head and pelvis protocols, which included iterative reconstruction, compared to the thorax protocol, which applied the FDK reconstruction (Figure 1). Moreover, only minor CT number drifts with a maximum median change of 32 HU observed 6 months after installation (Figure 2). Over the full period of 18 months, still no systematic drift was seen (Figure S5 in the supplementary material). Generally, the CBCT scans acquired for lung cancer patients reconstructed with FDK showed large dose differences between the planning CT and CBCT. The dose deviations were decreased for the lung cancer patients with iCBCT reconstruction, and a maximum dose deviation of ± 3% was observed (Figure 3). Thus, dose calculation for lung cancer patients benefits from the iCBCT reconstruction algorithm, even though the algorithm was invented for non‐moving tissues. For HN and pelvic cancer patients, the CBCTs reconstructed with iCBCT mostly showed DVH deviations below ± 1%.

Our findings align with several recent reports showing that HyperSight iCBCT reconstruction substantially reduces scatter and artifacts, yielding CBCT images with CT numbers that more closely approach fan‐beam CT than previous CBCT systems in phantom‐based studies.^14^, ^18^, ^21^, ^30^, ^31^ The inter‐scanner variability has previously been tested across HyperSight CBCT systems installed at different centers.^19^, ^32^ CT number stability was also found across the full treatment course for prostate cancer patients.^20^ Furthermore, other studies support the results presented in the current study, that the dose differences between fan‐beam CT and HyperSight CBCT are clinically acceptable.^15^, ^17^, ^20^ A study demonstrated that, compared with standard non‐HyperSight CBCT images, HyperSight CBCT raises the gamma pass rate into the clinically acceptable range in most cases.^9^

Two studies examined HyperSight on O‐ring linacs dose calculations in patients, using directly the planning CT‐based conversion curves, and reported average or median gamma pass rates (2%,2 mm) above 95%^17^, ^20^ and dose deviations of up to approximately 2% for pelvic cases and 4% for lung cases, excluding outliers.^17^ We did not assess this, since we used two distinct planning CT scanners, each with its own dedicated conversion curve. Calibrating the conversion curve for each specific system yields higher accuracy, since differences in detector design, tube voltage, and reconstruction algorithms between the CT and CBCT scanner affect CT numbers and thereby the conversion curves.^27^, ^33^, ^34^, ^35^, ^36^, ^37^ Figure S6 illustrates that the three CBCT protocols exhibit distinct conversion curves, driven primarily by differences in tube voltage and, to a lesser degree, reconstruction algorithms (iCBCT vs FDK). Other studies support the use of CBCT‐specific conversion curves.^31^, ^32^ The combination of low intra‐scanner variability and longitudinal stability of CT numbers for the HyperSight CBCT imaging panels on C‐arm linacs allows for the creation of accurate conversion curves for CBCT‐based dose calculation, and we found dose deviations of up to approximately 1% for pelvic cases and 3% for lung cases, excluding outliers.

The image quality of CBCT has generally been found to be worse compared to fan‐beam CT, which has resulted in the development of many correction mechanisms,^38^, ^39^, ^40^, ^41^ including deep learning synthetic CT generation.^12^, ^42^, ^43^ However, the increased image quality of the novel CBCT systems may eliminate the need to correct the CBCT image quality.^44^, ^45^ This is naturally an advantage, as the correction algorithms may, in some cases, slightly obscure the anatomy seen on the uncorrected CBCT image.^46^, ^47^ Further, it will lead to a small time saving, not having to perform the image quality correction.

In this study, we evaluated the CBCT systems installed on seven linacs in a single institution, but a total of eleven CBCT imaging panels were included in the assessment, since four CBCT imaging panels were replaced during the timeframe of the study. For several of the replaced CBCT detectors, no cause was found for the detector failure, and the failure could not be reproduced by the vendor. As shown in Figure S4 (supplementary material), rebooting the detector reduced the CT number drift. This easy ‘solution’ will be tested in the future if further issues are noticed. This clearly shows that continued QA of CT number stability is important to catch any CBCT issues.

We performed very careful measurements of the conversion curves. For each set of CBCT scans acquired, each insert was scanned individually, placed in the center of the phantom, surrounded only by HE Solid Water, to ensure the best CT number accuracy, and to mitigate streak artifacts caused by high‐density bone inserts, which could otherwise distort the CT numbers of surrounding inserts. This is in line with recommendations for calibration of fan‐beam CT conversion curves.^27^ Larger image artefacts would likely have arisen with peripheral insert placement; however, as the main aim of this study was to evaluate the accuracy of dose calculation based on the conversion curves, this scenario was not investigated. In a multi‐center study using the same phantom as in this study, all eleven phantom inserts, including four bone inserts, were scanned together.^19^ The SDs of the CT numbers were reported to be larger in their study, compared to our study. E.g. the SD for the CT number of HE Cortical Bone insert in the Head protocol was 110 HU, whereas in this study it was 16 HU for the measurements at installation and 18 HU for all measurements over the 18 months, while it was 43 HU for the thorax and 29 HU for the pelvis mode over the 18 months. This indicates the importance of optimal insert configurations, rather than placing several bone inserts together.

The low CT number variance across the CBCT imaging panels and over time allowed for a single conversion curve per CBCT protocol rather than per imaging panel. However, a single conversion curve cannot be used across the three protocols, due to the difference in tube voltage and reconstruction algorithm (note the different scales on the axes of the three plots in Figure S6 in the supplementary material).

## 5 CONCLUSION

This study evaluated CT number stability of HyperSight CBCT systems across imaging panels and time for CBCT systems installed on seven C‐arm linacs in the same institution and found it to be highly stable even after 18 months of daily clinical use, with median CT number variations within 32 HU, allowing for a single CT number to mass density conversion curve per CBCT protocol. When applying these conversion curves, the iterative reconstruction algorithm demonstrated high dose calculation accuracy compared to fan‐beam CT with median dose differences within ± 0.5% for HN and pelvic patients and within ± 1.5% for lung cancer patients, whereas the FDK reconstruction exhibited insufficient accuracy, with median dose differences up to ± 2.4% and outliers up to ‐18.0%. These results show the feasibility of using HyperSight CBCT images for dose calculation in clinical photon‐based radiotherapy routine.

## AUTHOR CONTRIBUTIONS

**Vicki Trier Taasti**: Formal analysis; methodology; software; visualization; writing—original draft; writing—review & editing. **Mai‐Britt Linaa**: Conceptualization; investigation; methodology; writing—review & editing. **Camilla Skinnerup Byskov**: Investigation; writing—review & editing. **Morten Bjørn Jensen**: Investigation; writing—review & editing. **Ditte Sloth Møller**: Funding acquisition; writing—review & editing. **Lone Hoffmann**: Conceptualization; funding acquisition; investigation; methodology; writing—review & editing.

## CONFLICT OF INTEREST STATEMENT

LH and DSM have a research agreement with Varian.

## Supporting information




**Supporting File**: acm270752‐sup‐0001‐SuppMat.pdf.
