# Supplementary material for: Quantitative stability of CT numbers in novel cone‐beam CT: An intra‐institution cross‐scanner and longitudinal analysis
Source: J Appl Clin Med Phys. 2026 Aug 24;27(9):e70752. doi: 10.1002/acm2.70752 (PMC13504163; doi:10.1002/acm2.70752)
Supplement: Supplementary file 1 [file ACM2-27-e70752-s001.pdf]

# Supplementary material

## Quantitative stability of CT numbers in novel cone-beam CT: An intra-institution cross-scanner and longitudinal analysis

**Table S1:** Prescription doses for head-and-neck cancer patients. The reference dose, selected as the maximal prescription dose, was used in the calculation of the relative dose differences (see Figure 3 in the main text). *Abbreviations*: CTV: clinical target volume; fx: fractions.

| Patient number | Reference dose | Prescription doses |
|---|---|---|
| Patient 1 | 66 Gy | CTV1: 66 Gy / 33 fx; CTV2: 60 Gy / 33 fx; CTV3: 50 Gy / 33 fx |
| Patient 2 | 68 Gy | CTV1: 68 Gy / 34 fx; CTV2: 60 Gy / 34 fx; CTV3: 50 Gy / 34 fx |
| Patient 3 | 60 Gy | CTV2: 60 Gy / 30 fx; CTV3: 50 Gy / 30 fx |
| Patient 4 | 66 Gy | CTV1: 66 Gy / 33 fx; CTV2: 60 Gy / 33 fx; CTV3: 50 Gy / 33 fx |
| Patient 5 | 66 Gy | CTV1: 66 Gy / 33 fx; CTV2: 60 Gy / 33 fx; CTV3: 50 Gy / 33 fx |
| Patient 6 | 68 Gy | CTV1: 68 Gy / 34 fx; CTV2: 60 Gy / 34 fx; CTV3: 50 Gy / 34 fx |
| Patient 7 | 32 Gy | CTV1: 32 Gy / 8 fx |
| Patient 8 | 68 Gy | CTV1: 68 Gy / 34 fx; CTV2: 60 Gy / 34 fx; CTV3: 50 Gy / 34 fx |
| Patient 9 | 68 Gy | CTV1: 68 Gy / 34 fx; CTV2: 60 Gy / 34 fx; CTV3: 50 Gy / 34 fx |
| Patient 10 | 68 Gy | CTV1: 68 Gy / 34 fx; CTV2: 60 Gy / 34 fx; CTV3: 50 Gy / 34 fx |

**Table S2:** Prescription doses for lung cancer patients, reconstructed with either FDK (left part) or with iCBCT (right part). *Abbreviations*: fx: fractions.

| Patient number (FDK) | Prescription dose (FDK) | Patient number (iCBCT) | Prescription dose (iCBCT) |
|---|---|---|---|
| Patient 1 | 66 Gy / 33 fx | Patient 1 | 45 Gy / 30 fx |
| Patient 2 | 45 Gy / 30 fx | Patient 2 | 66 Gy / 33 fx |
| Patient 3 | 66 Gy / 33 fx | Patient 3 | 66 Gy / 33 fx |
| Patient 4 | 66 Gy / 33 fx | Patient 4 | 66 Gy / 33 fx |
| Patient 5 | 60 Gy / 40 fx | Patient 5 | 60 Gy / 40 fx |
| Patient 6 | 66 Gy / 33 fx | Patient 6 | 66 Gy / 33 fx |
| Patient 7 | 50 Gy / 24 fx | Patient 7 | 66 Gy / 33 fx |
| Patient 8 | 60 Gy / 30 fx | Patient 8 | 66 Gy / 33 fx |
| Patient 9 | 45 Gy / 30 fx | Patient 9 | 66 Gy / 33 fx |
| Patient 10 | 66 Gy / 33 fx | Patient 10 | 50 Gy / 24 fx |
| Patient 11 | 50 Gy / 24 fx | Patient 11 | 60 Gy / 40 fx |

**Table S3:** Prescription doses for pelvic cancer patients. The reference dose, selected as the maximal prescription dose, was used in the calculation of the relative dose differences (see Figure 3 in the main text). *Abbreviations*: fx: fractions.

| Patient number | Diagnose | Reference dose | Prescription doses |
|---|---|---|---|
| Patient 1 | Bladder | 66 Gy | Target 1: 66 Gy / 33 fx; Target 2: 50 Gy / 33 fx |
| Patient 2 | Prostate | 50 Gy | Target 1+2: 50 Gy / 25 fx |
| Patient 3 | Prostate | 68 Gy | Target 1+2: 68 Gy / 34 fx; Target 3: 51 Gy / 34 fx |
| Patient 4 | Prostate | 78 Gy | Target 1: 56 Gy / 39 fx; Target 2+3: 78 Gy / 39 fx |
| Patient 5 | Prostate | 78 Gy | Target 1: 56 Gy / 39 fx; Target 2+3: 78 Gy / 39 fx |
| Patient 6 | Prostate | 46 Gy | Target 1+2+3: 46 Gy / 23 fx |
| Patient 7 | Prostate | 78 Gy | Target 1+3: 56 Gy / 39 fx; Target 2: 78 Gy / 39 fx |
| Patient 8 | Cervix | 45 Gy | Target 1: 45 Gy / 25 fx |
| Patient 9 | Bladder | 64 Gy | Target 1: 64 Gy / 32 fx; Target 2: 50 Gy / 30 fx |
| Patient 10 | Bladder | 64 Gy | Target 1: 64 Gy / 32 fx |
| Patient 11 | Bladder | 36 Gy | Target 1: 36 Gy / 6 fx |
| Patient 12 | Rectum | 50.4 Gy | Target 1+2: 50.4 Gy / 28 fx |
| Patient 13 | Anal | 60 Gy | Target 1: 60 Gy / 30 fx; Target 2: 48 Gy / 30 fx |
| Patient 14 | Rectum | 25 Gy | Target 1+2: 25 Gy / 5 fx |
| Patient 15 | Vulva | 64 Gy | Target 1: 64 Gy / 32 fx; Target 2: 51.2 Gy / 32 fx |

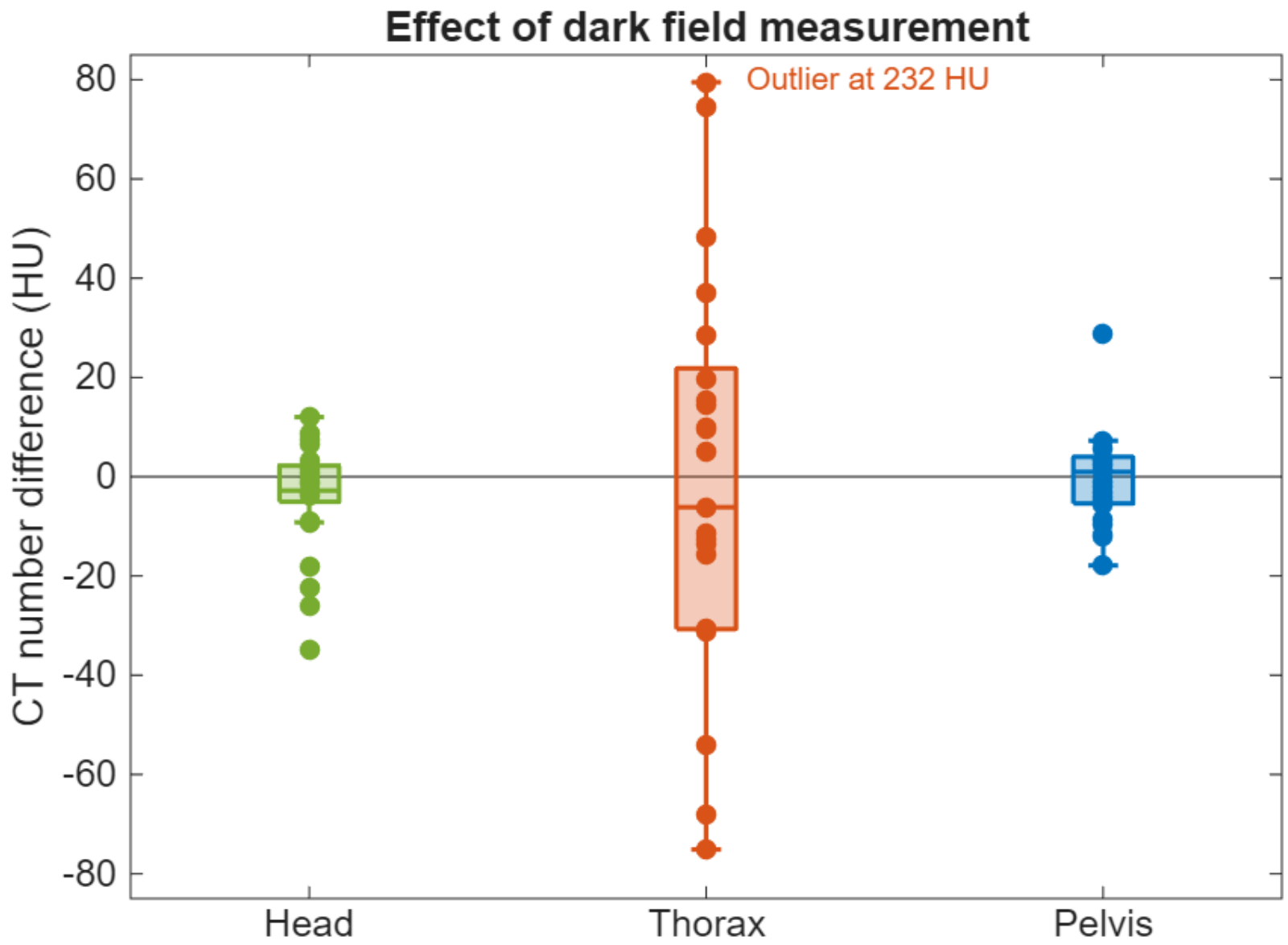


**Figure S1:** Effect of performing dark field measurement, visualized as the CT number difference for the HE Cortical Bone insert between a CBCT scan acquired before and after performing the dark field measurement.

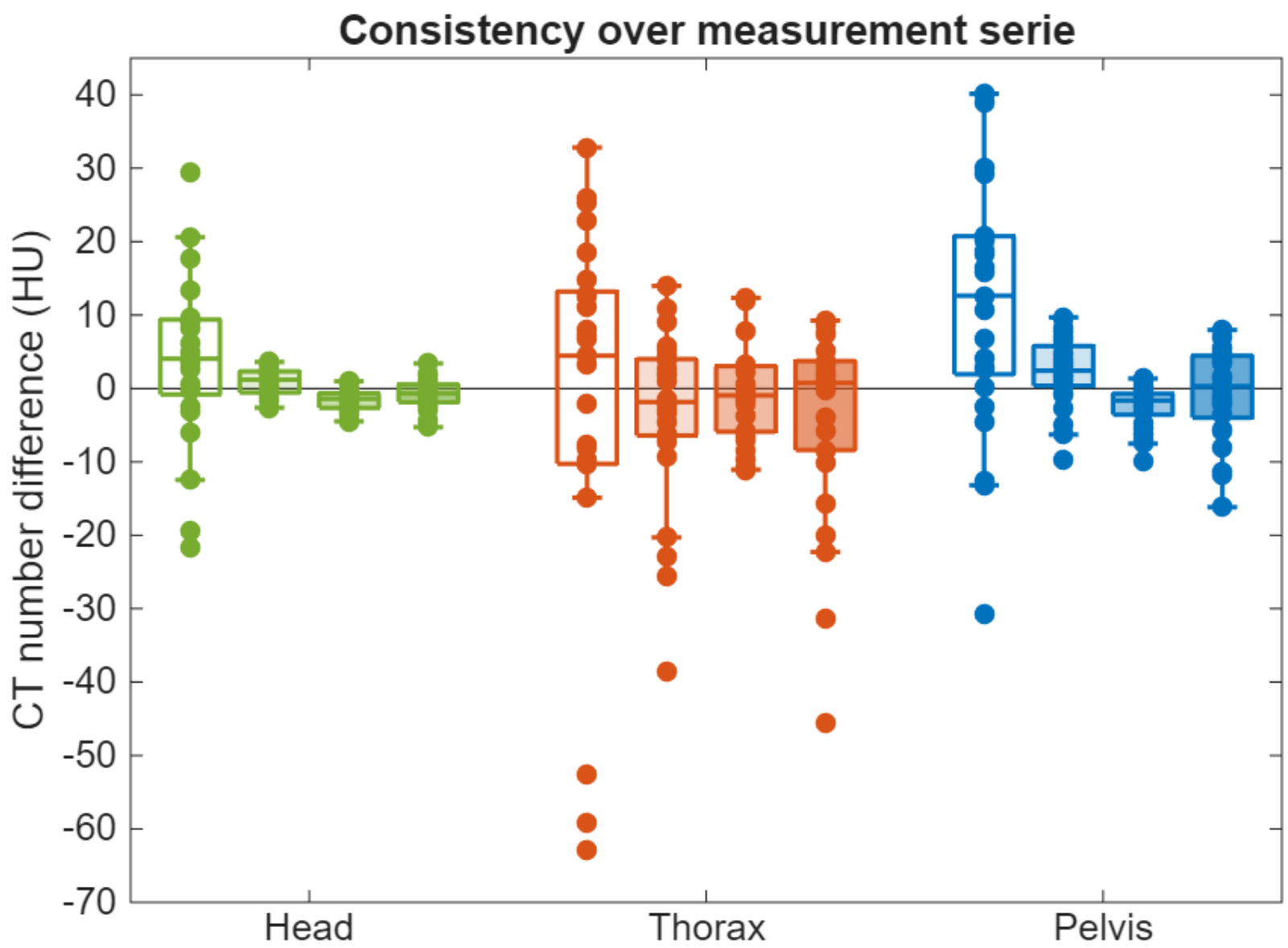


**Figure S2:** CT number difference for the HE Cortical Bone and the HE Solid Water inserts scanned at the beginning, middle and end of the measurement series, for assessment of potential CT number drift caused by detector after-glow. The evaluation was performed for each of the three CBCT scan protocols (head, thorax, and pelvis). For each scan protocol, the leftmost boxplot (no boxplot fill color) is the CT number differences for HE Cortical Bone, for a scan in the beginning of the measurement series (after performing the dark field) and a scan towards the end of the measurement series. The three next boxplots (with increasingly darker boxplot fill color) show CT number differences for the HE Solid Water insert, with the second boxplot showing CT number differences between a scan in the beginning of the measurement series (after performing the dark field) and a scan in the middle of the measurement series, the third boxplot shows the CT number differences between a scan in the middle of the measurement series and a scan at the end of the measurement series, and the fourth boxplot shows the CT number differences between a scan in the beginning of the measurement series and a scan at the end of the measurement series.

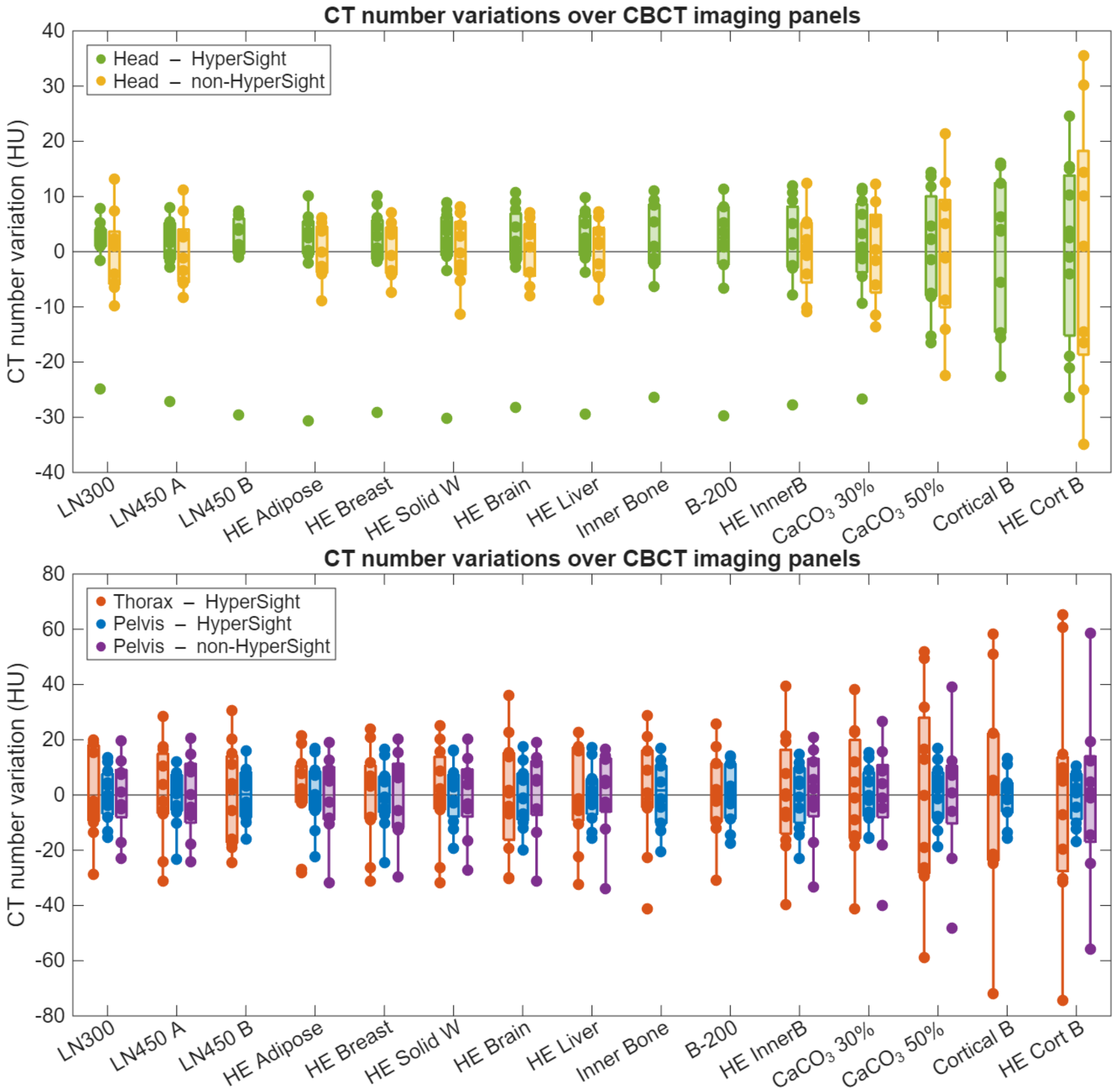


**Figure S3:** CT number variation across the CBCT imaging panels for the HyperSight (current CBCT version) and the predecessor non-HyperSight CBCT system. The data are shown as the mean across all imaging panels minus the actual CT number for the specific CBCT imaging panel for each of the 15 inserts.

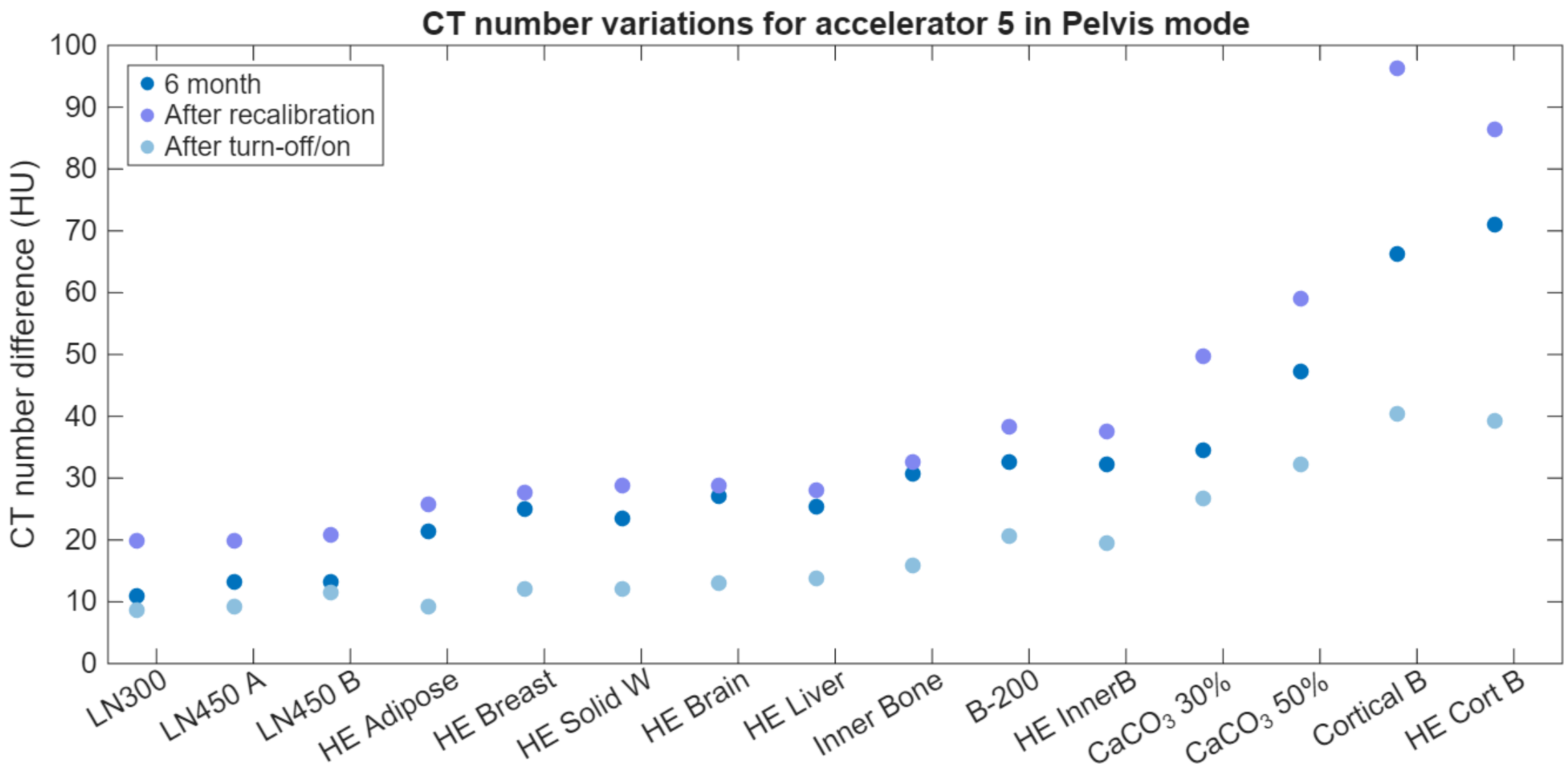


**Figure S4:** Test of outlier data points for the Pelvis mode on accelerator 5 at the 6-month evaluation (see lower part of Figure 2 in the main text).

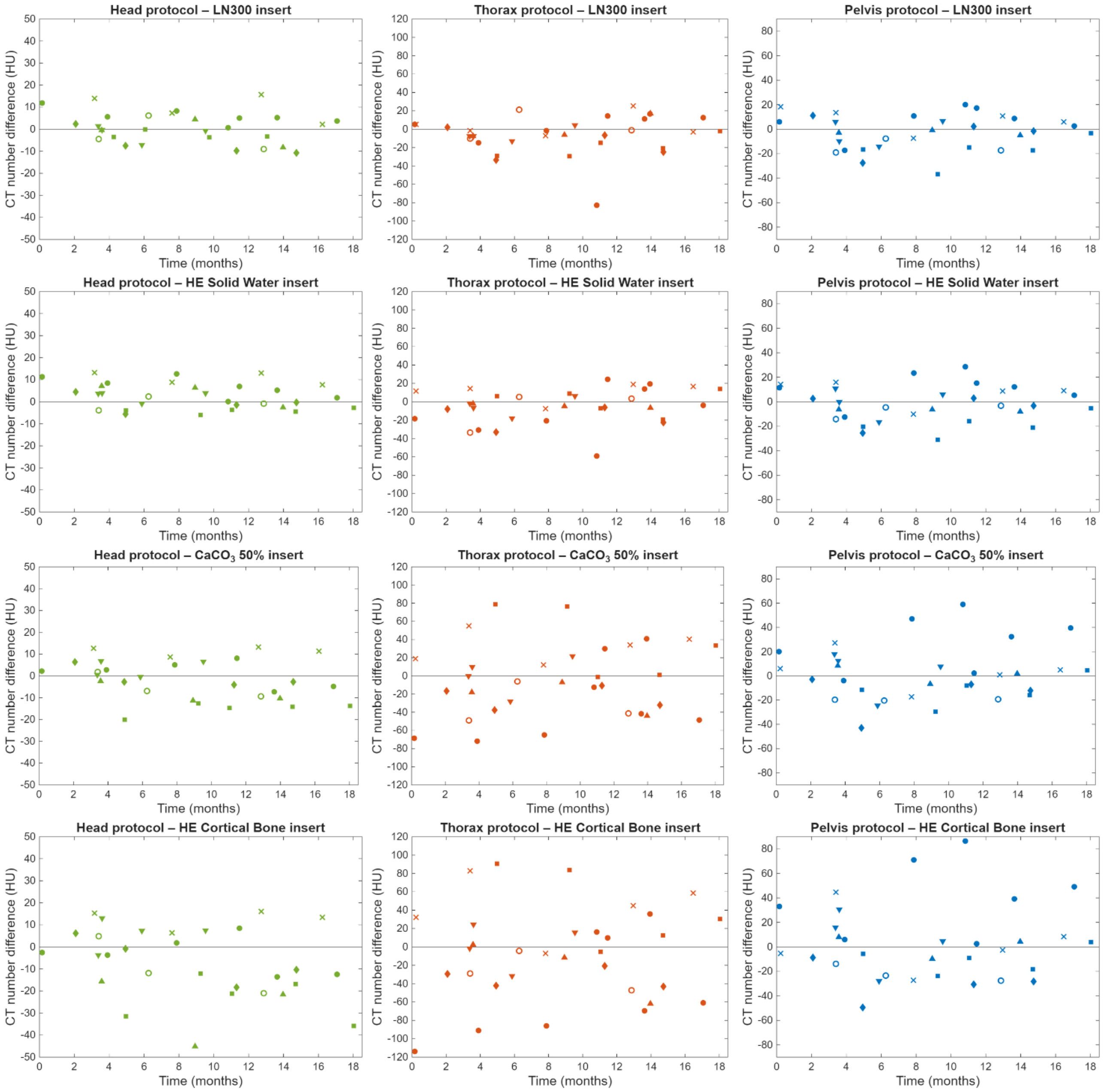


**Figure S5:** CT number variation as a function of time, with the CT number difference calculated against the measurement at installation (in case of imaging panel replacement, the measurement at installation of the replacement imaging panel was used as time zero). The different symbols indicate the different CBCT systems (at each of the seven linacs), to check that no trend was seen both across the CBCT scanners and for a given CBCT system. Note, the different y-axes between the plots for the three different CBCT protocols.

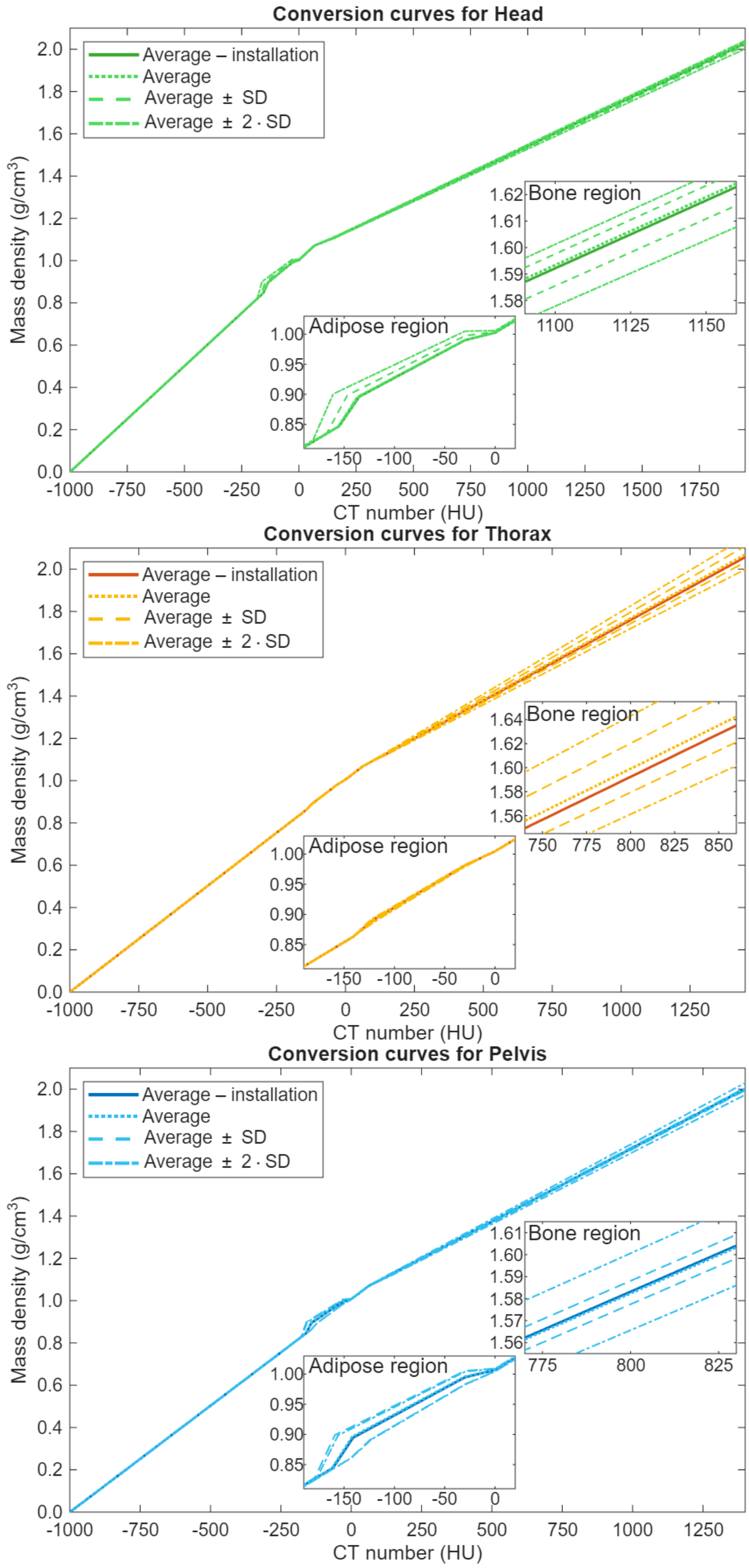


**Figure S6:** Mass density conversion curves created based on the CT numbers for the phantom inserts. For each CBCT mode (head, thorax, and pelvis), one conversion curve was created based on the average of the CT numbers for each insert across all CBCT imaging panels acquired at installation (full line), and one conversion curve was created based on the average of all CBCT acquisitions (at installation, as well as after three and six months) across all imaging panels. Moreover, conversion curves for the average ± 1 or 2 standard deviations (SD) were created, where the SDs were calculated including all acquisitions (all three time points and all CBCT imaging panels). Note, the x-axis scales differ between the conversion curves for the head and for the two others (due to the different tube voltages).
